# Prognostic and Immunological Potential of Ribonucleotide Reductase Subunits in Liver Cancer

**DOI:** 10.1155/2023/3878796

**Published:** 2023-01-20

**Authors:** Xin Yin, Kan Jiang, Ziyang Zhou, Hao Yu, Danfang Yan, Xingkang He, Senxiang Yan

**Affiliations:** ^1^Department of Radiation Oncology, The First Affiliated Hospital, College of Medicine, Zhejiang University, 79 Qingchun Road, Hangzhou, Zhejiang 310003, China; ^2^Department of Gastroenterology, Sir Run Run Shaw Hospital, Zhejiang University Medical School, Hangzhou 310016, China

## Abstract

**Background:**

Ribonucleotide reductase (RR) consists of two subunits, the large subunit RRM1 and the small subunit (RRM2 or RRM2B), which is essential for DNA replication. Dysregulations of RR were implicated in multiple types of cancer. However, the abnormal expressions and biologic functions of RR subunits in liver cancer remain to be elucidated.

**Methods:**

TCGA, HCCDB, CCLE, HPA, cBioPortal, and GeneMANIA were utilized to perform bioinformatics analysis of RR subunits in the liver cancer. GO, KEGG, and GSEA were used for enrichment analysis.

**Results:**

The expressions of RRM1, RRM2, and RRM2B were remarkably upregulated among liver cancer tissue both in mRNA and protein levels. High expression of RRM1 and RRM2 was notably associated with high tumor grade, high stage, short overall survival, and disease-specific survival. Enrichment analyses indicated that RRM1 and RRM2 were related to DNA replication, cell cycle, regulation of nuclear division, DNA repair, and DNA recombination. Correlation analysis indicated that RRM1 and RRM2 were significantly associated with several subsets of immune cell, including Th2 cells, cytotoxic cells, and neutrophils. RRM2B expression was positively associated with immune score and stromal score. Chemosensitivity analysis revealed that sensitivity of nelarabine was positively associated with high expressions of RRM1 and RRM2. The sensitivity of rapamycin was positively associated with high expressions of RRM2B.

**Conclusion:**

Our findings demonstrated high expression profiles of RR subunits in liver cancer, which may provide novel insights for predicting the poor prognosis and increased chemosensitivity of liver cancer in clinic.

## 1. Introduction

Liver cancer is one of the most common malignant diseases responsible for increasing cancer-related deaths globally [1]. Surgery, chemotherapy, and radiotherapy are the primary treatment strategies used for liver cancers [2]. During the last few years, targeted therapy and immunotherapy have achieved remarkable clinical responses and improved patient outcomes [3–5]. According to cancer statistics, the five-year survival rate for patients with advanced liver cancer is around 12% [1]. Therefore, there is an urgent need to investigate the potential molecular mechanisms and effective therapeutic strategies for liver cancer.

Ribonucleotide reductase (RR) is indispensable for reducing ribonucleotide diphosphates to deoxyribonucleotide diphosphates [6, 7]. There are two types of ribonucleotide reductases in humans, including RRM1–RRM2 and RRM1–RRM2B. RRM1–RRM2 play a crucial role in synthesizing deoxyribonucleoside triphosphates (dNTPs) for nuclear DNA replication, whereas RRM1–RRM2B provide dNTPs for nuclear and mitochondrial DNA replication [8, 9]. Emerging evidence has suggested that RR is implicated in the initiation and progression of multiple cancers [10–15]. Overexpression of RRM1 has been observed in lung cancers, sarcoma, and central nervous system cancers [16, 17]. Elevated RRM2 expression is associated with chemoresistance in pancreatic adenocarcinoma, whereas reduced expression can enhance gemcitabine-induced cytotoxicity [18]. High expression of RRM2B is also noted in lung cancers, melanoma, and oral carcinoma [8, 10, 19]. However, the comprehensive analysis and clinical significance of RR subunits remain to be elucidated.

In this study, we characterized the expression patterns and clinical significance of RR subunits in liver cancer. Potential biologic functions and immune cell infiltration of RR subunits were also evaluated. Our data thus provides a rationale for a novel liver cancer treatment strategy.

## 2. Methods

### 2.1. Data Source

For pan-cancer analysis, expression profiles of three RR subunits (RRM1, RRM2, and RRM2B) among more than 30 types of common human cancers in TCGA and GTEx datasets. Besides, HCCDB, A Database of Hepatocellular Carcinoma Expression Atlas (http://lifeome.net/database/hccdb/), was used to evaluate profiles of RRM1, RRM2, and RRM2B in liver cancer. Based on the Cancer Cell Line Encyclopedia (CCLE) dataset, we obtained expression levels of RR subunits from multiple liver cancer cell lines. All data included in our study was publicly available online. Utilizing the Human Protein Atlas (HPA) dataset, protein expressions of RRM1, RRM2, and RRM2B were compared between liver cancer and normal tissues. Genetic alterations of RRM1, RRM2, and RRM2B, including mutation and amplification, were obtained from cBioPortal (http://www.cbioportal.org). Functional assays and interactions of RRM1, RRM2, and RRM2B were performed by GeneMANIA (http://www.genemania.org).

### 2.2. Functional Enrichment and Immune Analyses

Gene Ontology (GO) annotation analysis, Kyoto Encyclopedia of Genes and Genomes (KEGG) pathway enrichment analysis, and gene set enrichment analyses (GSEA) of RR subunit-related genes were performed by the “clusterProfiler” R package. Single-sample gene set enrichment analyses (ssGSEA) was adopted to quantify the degree of tumor-infiltrating immune cells from RNA-seq data. The correlationship between RRM1, RRM2, and RRM2B expression and the level of different subsets of immune cell was evaluated by the Spearman correlation analysis.

### 2.3. Statistical Analysis

Wilcoxon rank-sum test or Student's *t*-test was used to compare statistical difference among two groups. Kaplan-Meier analysis was employed for visualization of survival difference, and survival difference was evaluated using log-rank test. Receiver operating characteristic (ROC) curves were established to evaluate the diagnostic and prognostic significance of RR subunits. The area under the ROC curve (AUC) estimated the magnitude of efficiency. Spearman's correlation coefficients were calculated to assess the relationship between RR subunits and immune microenvironment. All statistical analyses were completed by R software (version 3.6.3). A *p* value < 0.05 was defined as statistical significance.

## 3. Results

### 3.1. Differential Expression of RR Subunits in Liver and Pan-Cancers

**Figure 1:**
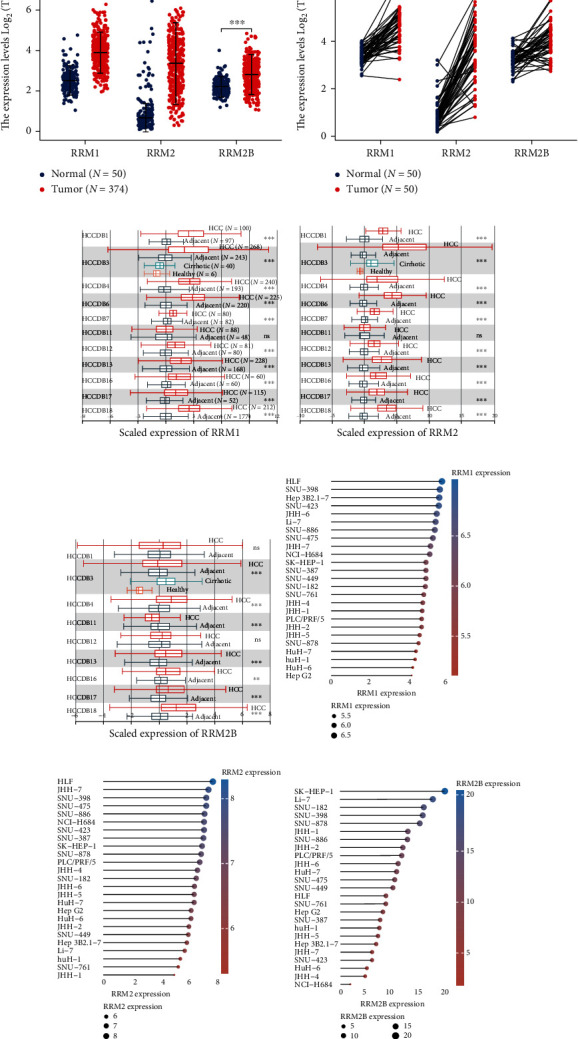
Expression of RR subunits in liver cancer and cancer cell lines. (a) mRNA expression levels of RRM1, RRM2, and RRM2B between liver cancer (tumor) and adjacent nonmalignant liver tissue (normal) in The Cancer Genome Atlas Liver Hepatocellular Carcinoma (TCGA-LIHC) cohort. (b) mRNA expression levels of RRM1, RRM2, and RRM2B in paired tumor and adjacent nonmalignant (normal) tissues from TCGA-LIHC cohort. (c) mRNA expression levels of RRM1 among various liver cancer cohorts in the Hepatocellular Carcinoma Expression Atlas Database (HCCDB). (d) mRNA expression levels of RRM2 among various liver cancer cohorts in the HCCDB. (e) mRNA expression levels of RRM2B among various liver cancer cohorts in the HCCDB. (f) mRNA expression levels of RRM1 in a variety of liver cancer cell lines from Cancer Cell Line Encyclopedia (CCLE). (g) mRNA expression levels of RRM2 in a variety of liver cancer cell lines from CCLE. (h) mRNA expression levels of RRM2B in a variety of liver cancer cell lines from CCLE. ^∗∗^*p* < 0.01 and ^∗∗∗^*p* < 0.001. The “ns” stands for “not significant”.

The expression profiles of RR subunits were compared in a variety of cancers. In most cancers, the mRNA expression of RRM1, RRM2, and RRM2B was generally increased (Figure S1). In TCGA cohort, the expression of RRM1, RRM2, and RRM2B was significantly higher in liver cancer (Figure 1(a)). These findings were also verified in paired normal liver cancer tissues (Figure 1(b)). We further utilized an integrated HCCDB dataset to compare the RR subunits. In multiple cohorts of HCCDB, RRM1, RRM2, and RRM2B were abnormally elevated in liver cancer tissues (Figures 1(c)–1(e)). In addition, high expression profiles of RRM1, RRM2, and RRM2B were observed in multiple liver cancer cell lines from the CCLE (Figures 1(f)–1(h)). Moreover, high levels of RRM1, RRM2, and RRM2B proteins were also based on the HPA dataset (Figures S2A-2C).

### 3.2. Association between Expression of RR Subunits and Clinicopathological Characteristics

**Figure 2:**
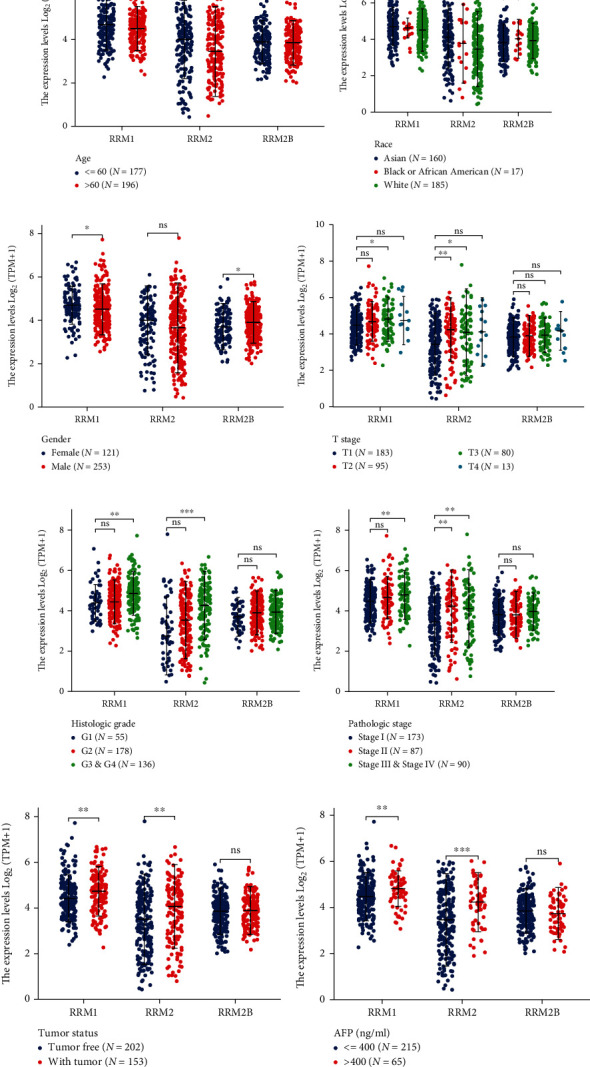
Correlation analysis of clinicopathological characteristics and expressions of RR subunits in liver cancer tissues. (a) Expressions of RRM1, RRM2, and RRM2B in different age groups (number of age < = 60 years: 177 and number of age > 60 years: 196). (b) Expressions of RRM1, RRM2, and RRM2B in different ethnic groups (number of Asian:160, number of Black or African American: 17, and number of White: 185). (c) Expressions of RRM1, RRM2, and RRM2B in different gender groups (number of female: 121 and number of male: 253). (d) Expressions of RRM1, RRM2, and RRM2B in different T stage groups (number of T1: 183, number of T2: 95, number of T3: 80, and number of T4: 13). (e) Expressions of RRM1, RRM2, and RRM2B in different grade groups (number of G1: 55, number of G2: 178, and number of G3 and G4: 136). (f) Expressions of RRM1, RRM2, and RRM2B in different stage groups (number of stage I: 173, number of stage II: 87, and number of stages III and IV: 90). (g) Expressions of RRM1, RRM2, and RRM2B in different tumor status groups (number of tumor-free: 202 and number of with tumor: 153). (h) Expressions of RRM1, RRM2, and RRM2B in different alpha-fetoprotein (AFP) protein level groups (number of AFP < = 400 ng/ml: 215 and number of AFP > 400 ng/ml: 65). ^∗^*p* < 0.05, ^∗∗^*p* < 0.01, and ^∗∗∗^*p* < 0.001. The “ns” stands for “not significant.” “Tumor-free” means that liver cancer does not continue to be present, indicating no progression of the original liver cancer. “With tumor” means the progression of the original disease.

To explore the clinical implications of RR subunits, we compared the differences of RRM1, RRM2, and RRM2B among clinicopathologic features, including age, race, gender, T stage, grade, stage, tumor status, and alpha-fetoprotein (AFP) level. High level of RRM1 mRNA was found to be associated with those who were young aged (<=60), female patients, T3 stage, poorly differentiated and undifferentiated (G3 and G4), advanced stage (stages III and IV), and high AFP levels (Figure 2). High level of RRM2 mRNA was associated with the Asian race, T2, T3 stage, poorly differentiated and undifferentiated (G3 and G4), advanced stage (stage II and stages III and IV), and high AFP levels (Figure 2). In the case of RRM2B, higher expression was only associated with males (Figure 2(c)).

### 3.3. Association between Expression of RR Subunits and Survival Outcomes

**Figure 3:**
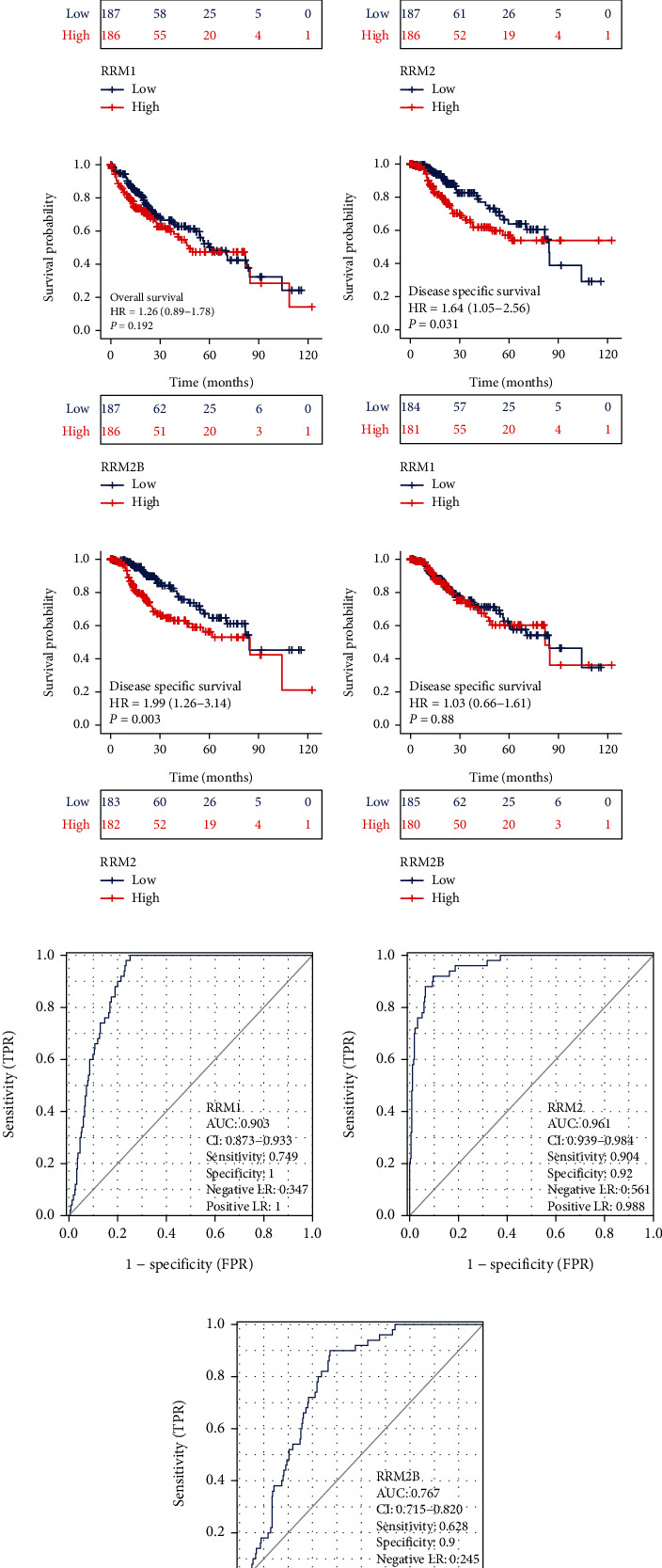
Prognostic impact of RRM1, RRM2, and RRM2B in TCGA-LIHC. (a) Kaplan-Meier overall survival analysis between low (*N* = 187) and high (*N* = 186) expression of RRM1. (b) Kaplan-Meier overall survival analysis between low (*N* = 187) and high (*N* = 186) expression of RRM2. (c) Kaplan-Meier overall survival analysis between low (*N* = 187) and high (*N* = 186) expression of RRM2B. (d) Kaplan-Meier disease-specific survival analysis between low (*N* = 184) and high (*N* = 181) expression of RRM1. (e) Kaplan-Meier disease-specific survival analysis between low (*N* = 183) and high (*N* = 182) expression of RRM2. (f) Kaplan-Meier disease-specific survival analysis between low (*N* = 185) and high (*N* = 180) expression of RRM2B. (g) The receiver operating characteristic (ROC) curve for the diagnosis of liver cancer based on RRM1. (h) The ROC curve for the diagnosis of liver cancer based on RRM2. (i) The ROC curve for the diagnosis of liver cancer based on RRM2B.

To evaluate the prognostic values of RR subunits, we adopted the Kaplan-Meier curves to analyze the correlation between the expression of RR subunits and survival outcomes. As shown in Figures 3(a) and 3(b), high levels of RRM1 were significantly correlated with poor overall survival (OS) (HR = 1.48, 95% CI, 1.04-2.09, *p* = 0.027) and disease-specific survival (DSS) (HR = 1.64, 95% CI, 1.05-2.56, *p* = 0.031) in the short term; however, they had better prognosis in the long term. As seen in Figures 3(c) and 3(d), high expression of RRM2 was significantly associated with poor OS (HR = 1.70, 95% CI, 1.20-2.41, *p* = 0.003) and DSS (HR = 1.99, 95% CI, 1.26-3.14, *p* = 0.003). In case of RRM2B, there was no significant association among OS (HR = 1.26, 95% CI, 0.89-1.78, *p* = 0.192) or DSS (HR = 1.03, 95% CI, 0.66-1.61, *p* = 0.88) (Figures 3(e) and 3(f)). Furthermore, we also explored the potential diagnostic value of RR subunits in diagnosing liver cancer. The ROC curve analysis demonstrated that the discriminative abilities of liver cancer for RRM1, RRM2, and RRM2B were 0.903 (95% CI: 0.873-0.933, Figure 3(g)), 0.961 (95% CI: 0.939-0.984, Figure 3(h)) and 0.767 (95% CI: 0.715-0.820, Figure 3(i)), respectively. Time-dependent ROC curves were adopted to compare the prognostic accuracy of RRM1, RRM2, and RRM2B in predicting the prognosis of liver cancer. The ROC showed that AUC values for the OS of RRM1, RRM2, and RRM2B were 0.673, 0.718, and 0.615, respectively, and AUC values for the DSS of RRM1, RRM2, and RRM2B were 0.741, 0.763, and 0.509, respectively (Figures S3A and 3B).

### 3.4. Genetic Alteration and Interaction Analyses of RR Subunits

**Figure 4:**
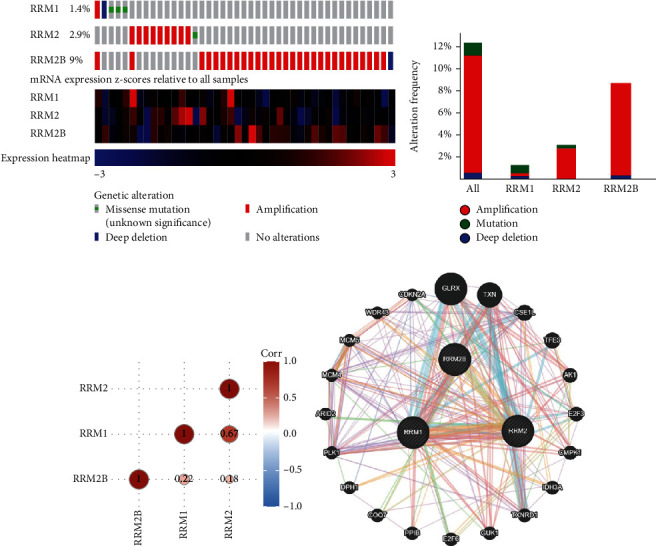
Mutation patterns and coexpression analyses of RR subunits. (a) Summary genetic alterations of RRM1, RRM2, and RRM2B in TCGA-LIHC (*N* = 370). (b) Frequency of different somatic alterations of RRM1, RRM2, and RRM2B in TCGA-LIHC. (c) Coexpression matrix of RRM1, RRM2, and RRM2B. (d) Interaction gene networks of RRM1, RRM2, and RRM2B, such as glutaredoxin (GLRX), thioredoxin (TXN), chromosome segregation 1-like (CSE1L), transcription factor binding to IGHM enhancer 3 (TFE3), adenylate kinase 1 (AK1), E2F transcription factor 3 (E2F3), cytidine/uridine monophosphate kinase 1 (CMPK1), isocitrate dehydrogenase (NAD(+)) 3 catalytic subunit alpha (IDH3A), thioredoxin reductase 1 (TXNRD1), guanylate kinase 1 (GUK1), E2F transcription factor 6 (E2F6), peptidylprolyl isomerase B (PPIB), coenzyme Q7, hydroxylase (COQ7), diphthamide biosynthesis 1 (DPH1), (polo-like kinase 1) PLK1, AT-rich interaction domain 2 (ARID2), MCM4, minichromosome maintenance complex component 5 (MCM5), WD repeat domain 43 (WDR43), and cyclin-dependent kinase inhibitor 2A (CDKN2A).

Genetic alterations of RR subunits among liver cancer patients were evaluated through the cBioPortal database. The RRM1, RRM2, and RRM2B were altered in 46 of 370 patients, accounting for 12.4% (Figure 4(a)). Alteration frequency of RRM1, RRM2, and RRM2B was 1.4%, 2.0%, and 9% (Figure 4(a)). For all RR subunits, gene amplification is the most common type of gene mutation. Usually, mutation is followed by deep deletion (Figure 4(b)). Correlation analysis also indicated that RRM1 and RRM2 had the highest correlation in liver cancer (Figure 4(c)). Using the GeneMANIA dataset, we identified RR subunit-associated molecules, such as glutaredoxin (GLRX), thioredoxin (TXN), chromosome segregation 1 like (CSE1L), transcription factor binding to IGHM enhancer 3 (TFE3), adenylate kinase 1 (AK1), E2F transcription factor 3 (E2F3), cytidine/uridine monophosphate kinase 1 (CMPK1), isocitrate dehydrogenase (NAD(+)) 3 catalytic subunit alpha (IDH3A), thioredoxin reductase 1 (TXNRD1), guanylate kinase 1 (GUK1), E2F transcription factor 6 (E2F6), peptidylprolyl isomerase B (PPIB), coenzyme Q7, hydroxylase (COQ7), diphthamide biosynthesis 1 (DPH1), (polo-like kinase 1) PLK1, AT-rich interaction domain 2 (ARID2), MCM4, minichromosome maintenance complex component 5 (MCM5), WD repeat domain 43 (WDR43), and cyclin-dependent kinase inhibitor 2A (CDKN2A) (Figure 4(d)).

### 3.5. Functional Enrichment Analysis of RR Subunits and RR-Associated Genes

**Figure 5:**
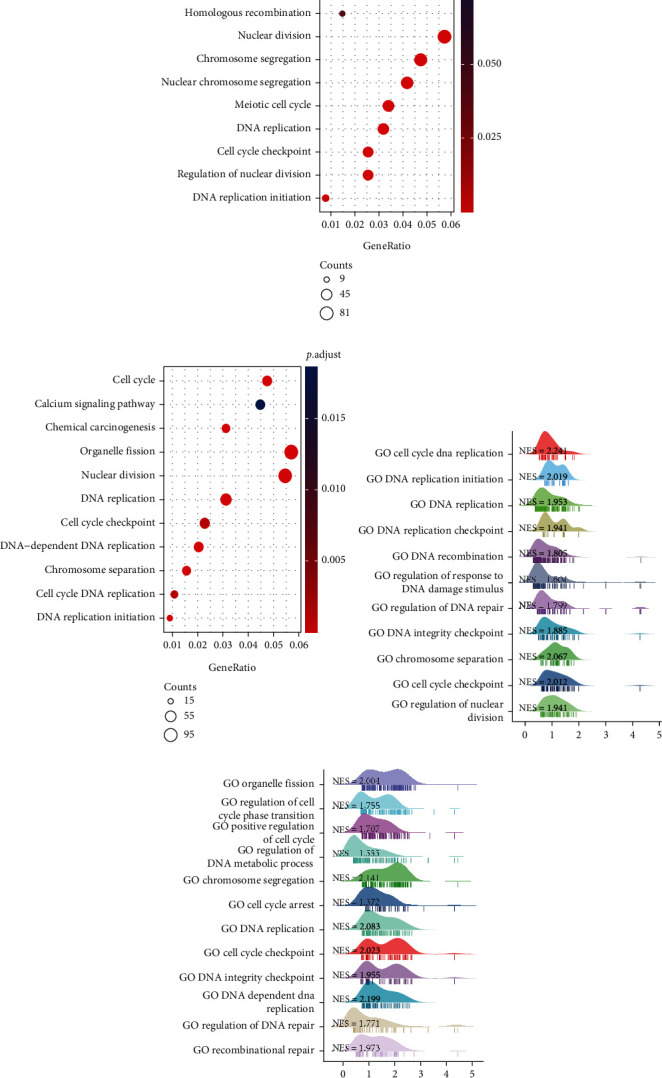
Functional enrichment analysis of RR subunits in liver cancer. (a) Gene Ontology (GO) and Kyoto Encyclopedia of Genes and Genomes (KEGG) enrichment results of RRM1-related genes (*N* = 374). (b) Ridge plot of GSEA results for high RRM1 expression. (c) GO and KEGG enrichment results of RRM2-related genes. (d) Ridge plot of GSEA results for high RRM2 expression. “NES,” normalized enrichment score (a significant positive NES value indicates that members of the gene set tend to appear at the top of the ranked transcriptome data.).

To further explore the potential biological functions of RR subunits, differentially expressed genes between high and low expression of RR subunits were analyzed by GO and KEGG enrichment analyses. As shown in Figures 5(a) and 5(b), biological functions and KEGG pathways for RRM1 and RRM2 were predominantly enriched in organelle fission, nuclear division, mitotic nuclear division, and DNA replication initiation. The RRM1-related genes were further analyzed by GSEA to identify several key functions, such as cell cycle DNA replication and its initiation, chromosome separation, cell cycle checkpoint, and regulation of DNA repair (Figure 5(c)). The RRM2-related genes were also analyzed by GSEA to identify several key functions, such as organelle fission, positive regulation of cell cycle, cell cycle arrest, DNA integrity checkpoint, and regulation of DNA metabolic process (Figure 5(d)).

### 3.6. Immune Cell Infiltration of RR Subunits

**Figure 6:**
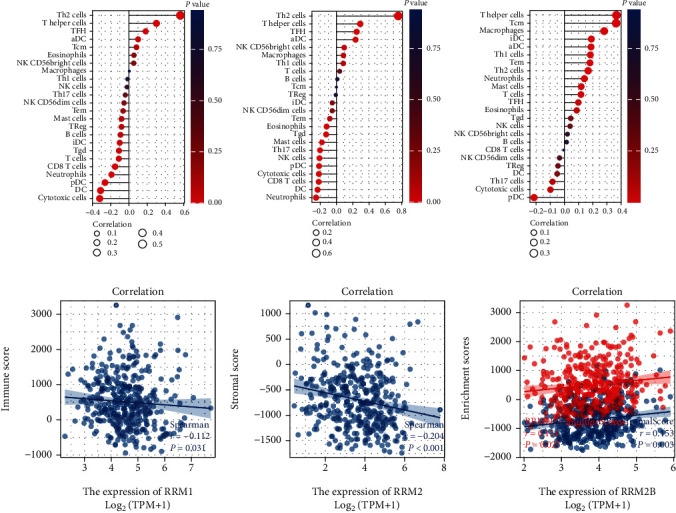
Immune analysis of RR subunits in liver cancer. (a) The correlation between RRM1 expression and profiles of immune infiltrating cells. (b) The correlation between RRM2 expression and profiles of immune infiltrating cells. (c) The correlation between RRM2B expression and profiles of immune infiltrating cells. (d) The correlation between RRM1 expression and immune score. (e) The correlation between RRM2 expression and stromal score. (f) The correlation between RRM2B expression and immune and stromal score. Abbreviation: TH2: T helper 2; TFH: T follicular helper cells; aDC: activated dendritic cell; Tcm: central memory T cell; TH1: T helper 1; TH17: T helper 17; NK: natural killer cells; Tem: effector memory T cell; pDC: plasmacytoid dendritic cell; DC: dendritic cell.

Immune cells, an important part of the tumor microenvironment (TME), contribute to the progression of tumors. Therefore, we evaluated the relationship between the expression of RR subunits and the infiltration of various immune cells. We found that RRM1 expression was positively associated with T helper 2 (Th2) cells and negatively associated with cytotoxic cells and dendritic cells (DCs) (Figure 6(a)). Similarly, RRM2 expression was positively associated with Th2 cells and negatively associated with neutrophils and dendritic cells (DCs) (Figure 6(b)). Moreover, RRM2B expression was positively associated with Th cells and central memory T cell (Tcm) and negatively associated with plasmacytoid dendritic cell (pDC) (Figure 6(c)). Furthermore, we demonstrated that stroma score was positively correlated with RRM2B expression and negatively correlated with RRM2 expression. However, the immune score was positively correlated with RRM2B expression and negatively correlated with RRM1 expression (Figures 6(d)–6(f)).

### 3.7. Correlation between Expression of RR Subunits and Chemotherapeutics

To assess the potential impact of the RR subunits on chemotherapy of liver cancer, we evaluated the correlations between the expression levels of RRM1, RRM2, and RRM2B and multiple drugs. The high expression of RRM1 was positively associated with nelarabine and 6-thioguanine (Figures S4 and B) while negatively associated with denileukin diftitox ontak (Figure S4C). The high expression of RRM2 was positively associated with nelarabine (Figure S4D). In the case of RRM2B, the high expression was positively associated with rapamycin (Figure S4E) while negatively associated with decitabine and docetaxel (Figures S4F and S4G).

## 4. Discussion

RR consists of two subunits, RRM1 and RRM2 or RRM2B, which play key role in the regulation of deoxyribonucleoside triphosphate (dNTP) biosynthesis [20, 21]. Since dNTP production is essential for maintaining DNA replication fidelity and genomic integrity, dysregulation of RRs leads to tumorigenic transformation, cancer proliferation, and metastasis [22, 23]. A growing body of evidence implicates that elevated expression of RR subunits is a characteristic of various cancers. We validated these findings in pan-TCGA datasets. Importantly, we demonstrated that RRM1 and RRM2 were not only highly expressed in liver cancer patients but also multiple liver cancer cell lines, consistent with previous studies [24, 25]. In the current study, we also found that the expression of RRM2B was increased in liver cancer and related cell lines, contradictory to a previous study [26]. Tian et al. reported that loss of RRM2B in liver cancer was negatively associated with metastasis, and RRM2B inhibited cell migration through Egr-1/PTEN/Akt1 pathway [26]. Since the population in the current study mainly came from western countries, further studies are needed to validate the current findings. RRM1 and RRM2 are good predictors to distinguish cancer tissue from normal liver tissue based on TCGA cohort. As we all know, serum AFP is the currently available diagnostic test for liver cancer [27]. In the same cohort, the discriminative ability of liver cancer for AFP was 0.502 (95% CI: 0.418-0.586), indicating that RRM1 and RRM2 had great potential to be diagnostic biomarkers for liver cancer. Compared with AFP (positive likelihood ratio: 0.206 and negative likelihood ratio: 0.985), both RRM1 and RRM2 had greater positive likelihood ratios and less negative likelihood ratios, suggested that they might have a great diagnostic value for liver cancer. Clinical relevance of RR subunits also showed that high RRM1 and RRM2 expressions were associated with advanced stage and poorly differentiated status, while no significant correlation was observed between RRM2B expression and clinicopathological characteristics. Survival analysis indicated that high levels of RRM1 and RRM2 were significantly correlated with poor OS and DSS. It should be noted that patients with higher expression of RRM1 presented better OS and DSS in the long term. For RRM2B, there was no significant correlation between RRM2B expression and OS or DSS. These data indicated that the function of RRM2B may vary in different populations.

Using the GeneMANIA database, we also identified RRM1-, RRM2-, and RRM2B-related molecules, which were also involved with liver cancer. Functional enrichment analysis, including GO, KEGG pathway, and GSEA, revealed that nuclear division, DNA replication initiation, DNA repair, and organelle fission were significantly enriched when RRM1 and RRM2 were highly expressed, consistent with previous studies [28–30]. In the case of RRM2B, its pathways are enriched in the extracellular matrix organization, the stress response to the metal ion, and collagen metabolic process pathways, different from the functions of RRM1 and RRM2 [28].

Recent studies have demonstrated that upregulation of RRM1 has been observed in various cancers, including liver cancer [24, 31–33]. The high expression of RRM1 was associated with resistance to DNA-damaging platinum drugs, leading to worse outcomes [34, 35]. However, RRM1 was also reported to have an inhibitory effect on the occurrence, invasion, and metastasis of lung cancer [36]. RRM2 was considered an oncoprotein that promotes the proliferation, invasion, and metastasis of multiple cancers [37–39].

TME is important for tumor progression and recurrence. Immune cells and stromal cells contribute to the biological behavior of the tumor. The relationship between the expression of RR subunits and immune infiltration remains unclear. Our analysis firstly demonstrated that RRM1 was negatively correlated with immune score and RRM2 was positively correlated with stroma score. RRM2B was positively correlated with immune score and stroma score. Furthermore, we found that RRM1 expression was positively associated with Th2 cells and negatively associated with cytotoxic cells and DCs. RRM2 expression was positively associated with Th2 cells and negatively associated with neutrophils and DCs. RRM2B expression was positively associated with T helper cells and Tcm and negatively associated with pDC. Our data indicated the important role of RR subunits in the TME of liver cancer. Considering the key role of RR in dNTP homeostasis, RR could be a potential therapeutic target. Prediction of chemotherapy sensitivity analysis indicated that high expression of RRM1 and RRM2 was associated with increased sensitivity to nelarabine, which could inhibit DNA synthesis. High expression of RRM2B was associated with reduced sensitivity to docetaxel and decitabine. Therefore, these data provided new insights for developing novel RR inhibitors.

On the other hand, our study has several limitations. All data included in our study were obtained from online databases. Further clinical and experimental studies are required to verify our findings and explore the potential mechanisms.

In conclusion, we systematically analyzed the expression and genetic alteration of RR subunits in liver cancer. The high expression of RRM1 and RRM2 is associated with a worse prognosis in patients with liver cancer.

## Data Availability

The datasets supporting the conclusions of this article are included within the article.

## Abbreviations

AFP: Alpha-fetoprotein
RR: Ribonucleotide reductase
TCGA: The Cancer Genome Atlas
LIHC: Liver hepatocellular carcinoma
HCCDB: A Database of Hepatocellular Carcinoma Expression Atlas
CCLE: The Cancer Cell Line Encyclopedia
HPA: The Human Protein Atlas
GO: Gene Ontology
KEGG: Kyoto Encyclopedia of Genes and Genomes
GSEA: Gene set enrichment analyses
ssGSEA: Single-sample gene set enrichment analyses
ROC: Receiver operating characteristic
OS: Overall survival
DSS: Disease-specific survival
dNTPs: Deoxyribonucleoside triphosphates.

## Supplementary Materials

Supplementary Materials These supplementary materials include four supplemental figures and one certificate of language editing of the manuscript. Figure S1 shows the expression of RRM1, RRM2, and RRM2B in various types of cancer. Figure S2 shows the protein expression of RRM1, RRM2, and RRM2B and the receiver operating characteristic curve for the diagnosis of liver cancer based on RRM1, RRM2, and RRM2B. Figure S3 shows the time-dependent receiver operating characteristic curve for prognosis of liver cancer based on RRM1, RRM2, and RRM2B. Figure S4 shows the correlation analysis of RR subunits with potential chemosensitivity.
