# Supplementary material for: Prognostic and Immunological Potential of Ribonucleotide Reductase Subunits in Liver Cancer
Source: Oxid Med Cell Longev. 2023 Jan 20;2023:3878796. doi: 10.1155/2023/3878796 (PMC9883104; doi:10.1155/2023/3878796)
Supplement: Supplementary Materials — These supplementary materials include four supplemental figures and one certificate of language editing of the manuscript. Figure S1 shows the expression of RRM1, RRM2, and RRM2B in various types of cancer. Figure S2 shows the protein expression of RRM1, RRM2, and RRM2B and the receiver operating characteristic curve for the diagnosis of liver cancer based on RRM1, RRM2, and RRM2B. Figure S3 shows the time-dependent receiver operating characteristic curve for prognosis of liver cancer based on RRM1, RRM2, and RRM2B. Figure S4 shows the correlation analysis of RR subunits with potential chemosensitivity. [file 3878796.f1.zip › language editing.pdf]

CERTIFICATE OF LANGUAGE EDITING

The English writing of the following manuscript was carefully edited by a native English speaker.

Manuscript Information

ID	LE202210240433
Editing date	2022-10-27
Title	Identification of ribonucleotide reductase subunits as independent prognostic and chemosensitivity implications in liver cancer
Corresponding author	Senxiang Yan
Language writing before editing	□ Very poor □ Poor □ Fair ■ Good □ Very good □ Excellent
Recommendation after language editing	□ Submitting to target journal directly<br>■ Submitting to target journal after minor revision<br>□ Re-editing required after major revision<br>□ Not suitable for publication

Overview comments	Thank you for using Editorbar service! The manuscript was written with good command of English. However, there are minor changes in the grammar and sentence constructions which have been changed. Moreover, it would be better if the author provides abstract and materials and methods for better understanding. Please make appropriate corrections and can be submitted to the journal. Thank you!

Edited by

Prajakta T.

Senior Editor

Queen's University Belfast

Language Editing

Certificate Issued by

Dr. Jason Qee

Editor in Chief

Editorbar Language Editing, Beijing, China

runse@editorbar.com www.editorbar.com

Certificate link: www.editorbar.com/order/cert/LE202210240433
