# Supplementary material for: Prognostic and Immunological Potential of Ribonucleotide Reductase Subunits in Liver Cancer
Source: Oxid Med Cell Longev. 2023 Jan 20;2023:3878796. doi: 10.1155/2023/3878796 (PMC9883104; doi:10.1155/2023/3878796)
Supplement: Supplementary Materials — These supplementary materials include four supplemental figures and one certificate of language editing of the manuscript. Figure S1 shows the expression of RRM1, RRM2, and RRM2B in various types of cancer. Figure S2 shows the protein expression of RRM1, RRM2, and RRM2B and the receiver operating characteristic curve for the diagnosis of liver cancer based on RRM1, RRM2, and RRM2B. Figure S3 shows the time-dependent receiver operating characteristic curve for prognosis of liver cancer based on RRM1, RRM2, and RRM2B. Figure S4 shows the correlation analysis of RR subunits with potential chemosensitivity. [file 3878796.f1.zip › SI_revised.docx]

**Supplemental figures**

**Figure S1. Expression of RRM1, RRM2, RRM2B in various types of cancers.**


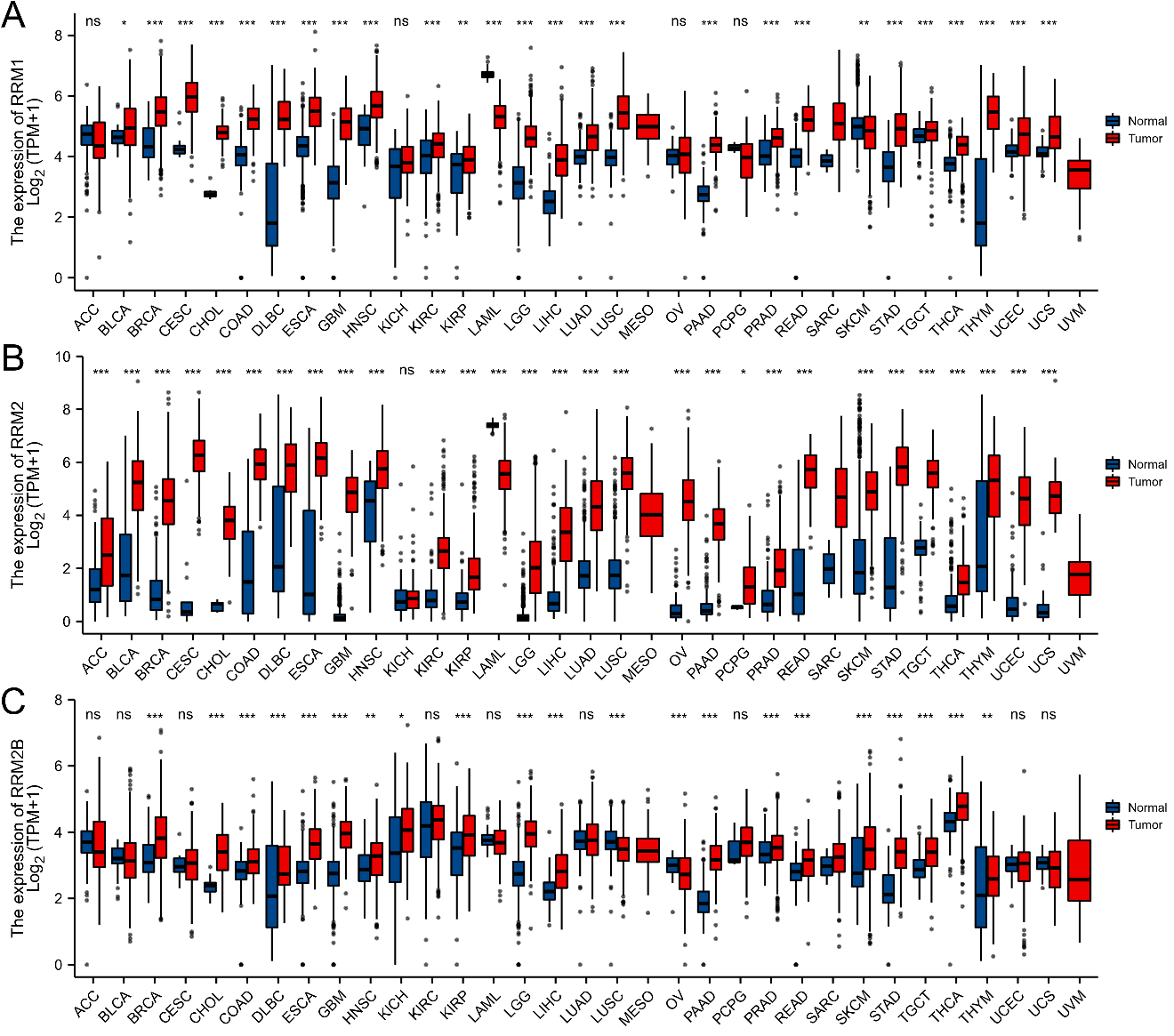


(A) mRNA expression levels of RRM1 in various cancer types in The Cancer Genome Atlas (TCGA, Number of tumor tissue: 373, Number of ). (B) mRNA expression levels of RRM2 in various cancer types in TCGA. (A) mRNA expression levels of RRM2B in various cancer types in TCGA. *p < 0.05, **p < 0.01, ***p < 0.001. The “ns” stands for “not significant”. Abbreviation: ACC, Adrenocortical carcinoma; BLCA, Bladder Urothelial Carcinoma; BRCA, Breast invasive carcinoma; CESC, Cervical squamous cell carcinoma and endocervical adenocarcinoma; CHOL, Cholangio carcinoma; COAD, Colon adenocarcinoma; DLBC, Lymphoid Neoplasm Diffuse Large B-cell Lymphoma; ESCA, Esophageal carcinoma; GBM, Glioblastoma multiforme; HNSC Head and Neck squamous cell carcinoma; KICH, Kidney Chromophobe; KIRC, Kidney renal clear cell carcinoma; KIRP, Kidney renal papillary cell carcinoma; LAML Acute Myeloid Leukemia; LGG, Brain Lower Grade Glioma; LIHC, Liver hepatocellular carcinoma; LUAD, Lung adenocarcinoma; LUSC, Lung squamous cell carcinoma; MESO, Mesothelioma; OV, Ovarian serous cystadenocarcinoma; PAAD, Pancreatic adenocarcinoma; PCPG, Pheochromocytoma and Paraganglioma; PRAD, Prostate adenocarcinoma; READ, Rectum adenocarcinoma; SARC, Sarcoma; SKCM, Skin Cutaneous Melanoma; STAD, Stomach adenocarcinoma; TGCT, Testicular Germ Cell Tumors; THCA, Thyroid carcinoma; THYM, Thymoma; UCEC, Uterine Corpus Endometrial Carcinoma; UCS, Uterine Carcinosarcoma; UVM, Uveal Melanoma

**Figure S2. Protein expression of RRM1, RRM2, RRM2B and the receiver operating characteristic curve for the diagnosis of liver cancer based on RRM1, RRM2, RRM2B**

**
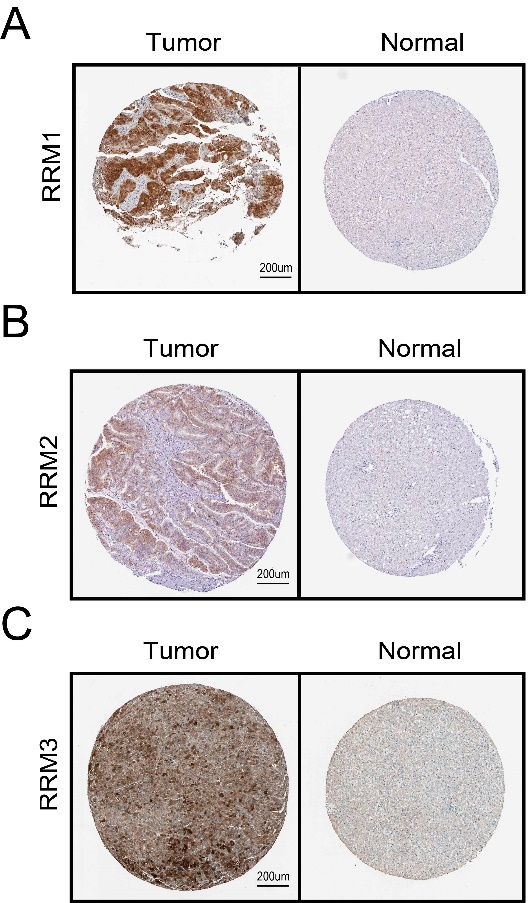
**

(A) The protein expression level of RRM1 in liver cancer from the Human Protein Atlas (HPA). (B) The protein expression level of RRM2 in liver cancer (HPA). (C) The protein expression level of RRM2B in liver cancer (HPA).

**Figure S3. Time-dependent receiver operating characteristic curve for prognosis of liver cancer based on RRM1, RRM2, RRM2B**


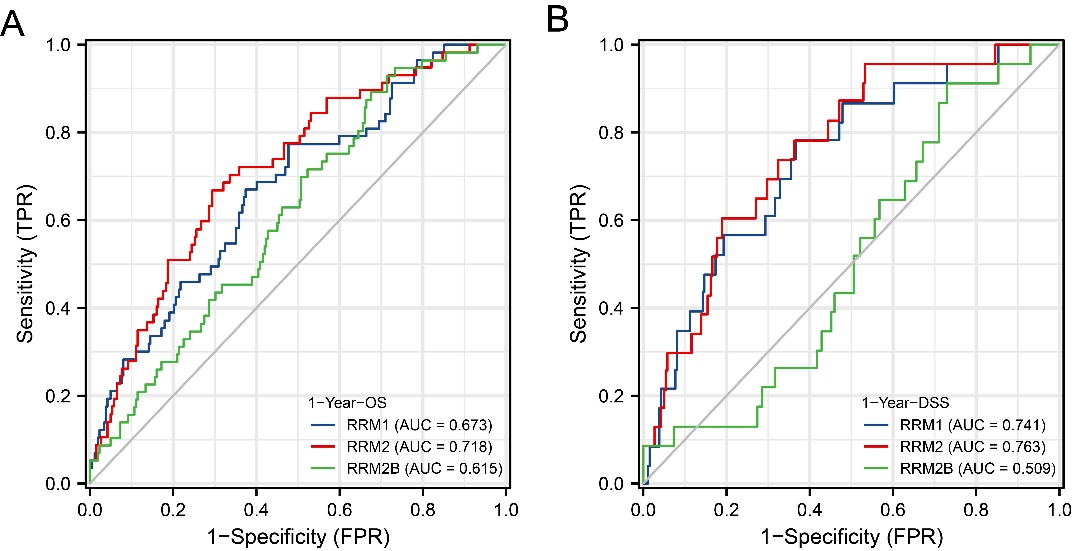


(A) Time-dependent receiver operating characteristic (ROC) curves at 1-year overall survival for RRM1, RRM2, RRM2B. (B) Time-dependent ROC curves at 1-year disease specific survival for RRM1, RRM2, RRM2B.

**Figure S4. The correlation analysis of RR subunits with potential chemosensitivity.**

**
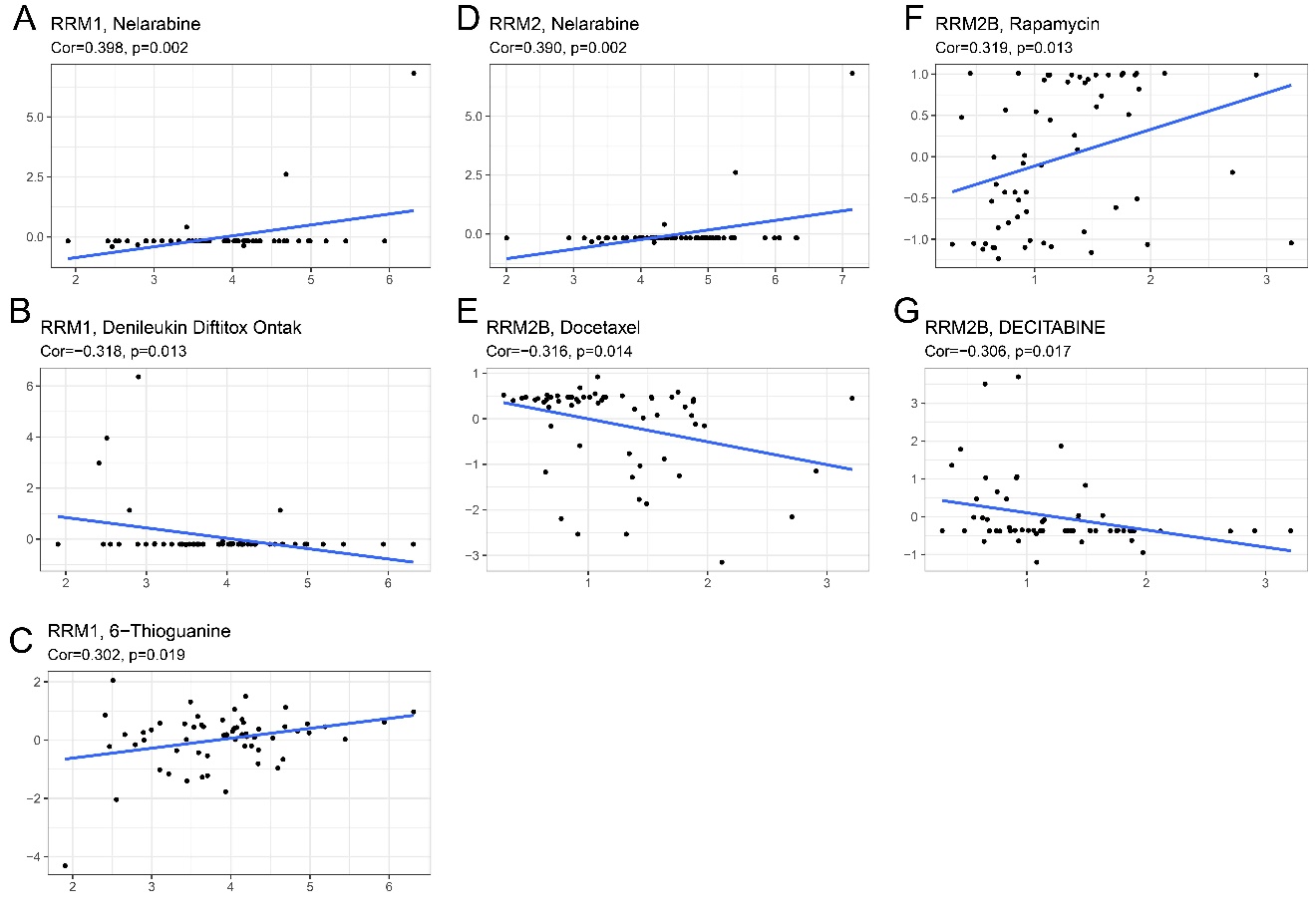
**

(A) RRM1 and Nelarabine. (B) RRM1 and Denileukin Diftitox Ontak. (C) RRM1 and 6-Thioguanine. (D) RRM2 and Nelarabine. (E) RRM2B and Docetaxel. (F) RRM2B and Rapamycin. (G) RRM2B and DECITABINE.
